# How to enhance prediction of clinical outcomes in poor responders: integrating high-specific assays for anti-mullerian hormone with antral follicle count

**DOI:** 10.3389/fendo.2025.1654365

**Published:** 2025-09-29

**Authors:** Laura Melado, Ajay Kumar, Bhanu Kalra, Erkan Kalafat, Barbara Lawrenz, Human Fatemi

**Affiliations:** ^1^ IVF Department, ART Fertility Clinics, Abu Dhabi, United Arab Emirates; ^2^ Scientific Department, Ansh Labs, Webster, TX, United States; ^3^ Division of Reproductive Endocrinology and Infertility, Koc University School of Medicine, Istanbul, Türkiye; ^4^ Department of Reproductive Medicine, UZ Ghent, Ghent, Belgium

**Keywords:** AMH isoforms, AFC, low ovarian reserve, ovarian response, AMH ELISA assays

## Abstract

**Introduction:**

Anti-Müllerian Hormone (AMH) and antral follicle count (AFC) are commonly used markers of ovarian reserve, yet their predictive accuracy in patients with low ovarian reserve remains limited. This study aimed to evaluate whether high-specific AMH assays targeting distinct molecular isoforms enhance the prediction of oocyte yield following ovarian stimulation (OS).

**Methods:**

A prospective observational study was conducted from February 2019 to December 2021 in a tertiary fertility center, including 72 women with low ovarian reserve (AMH <1.1ng/mL). On cycle day 2/3 before OS, Antral Follicle Count (AFC) and serum FSH, LH, estradiol, progesterone, and AMH levels were measured with the Elecsys assay (Roche). Frozen serum samples were analyzed with four high-specific AMH assays (AnshLabs, Texas): AL-196, AL-124, AL-105, and AL-133. Correlations were examined between AMH assays, AFC, and OS outcomes.

**Results:**

Patients’ median age was 39 years, with AFC of 5.5 and median AMH-Elecsys of 0.64 ng/mL. All AMH assays correlated significantly with AFC and stimulation outcomes. The AL-196 assay showed the highest correlation with the number of follicles, cumulus-oocyte complexes (COCs), and metaphase II (MII) oocytes. Models combining AFC and AMH assays were strong predictors of COCs and MII oocytes, with AFC+AL-196 offering the best predictive value (Adjusted R2 = 0.474 for COCs and 0.485 for MII, p<0.001).

**Conclusion:**

High-specific AMH assays using linear-epitope antibodies improve the accuracy of predicting oocyte yield in women with low ovarian reserve, thereby enabling more precise counselling and supporting personalized ovarian stimulation strategies.

**Clinical trial registration:**

NCT03826888.

## 1 Introduction

In the past decades, AMH and antral follicle count (AFC) have gained recognition as trusted biomarkers for assessing ovarian reserve and predicting response to ovarian stimulation (1, 2). However, clinicians frequently observe that AMH and AFC are discordant, even when performed during the early follicular phase in the same center (3). In such circumstances, clinicians are concerned about the non-alignment of the markers and deliberate on which to trust. This issue becomes critical when considering patients with expected poor ovarian response, as results may involve critical decisions on whether or not to proceed with OS, for whom clinical counselling should be as accurate as possible. This is where the understanding of the AMH physiology becomes particularly relevant.

**Figure 1:**
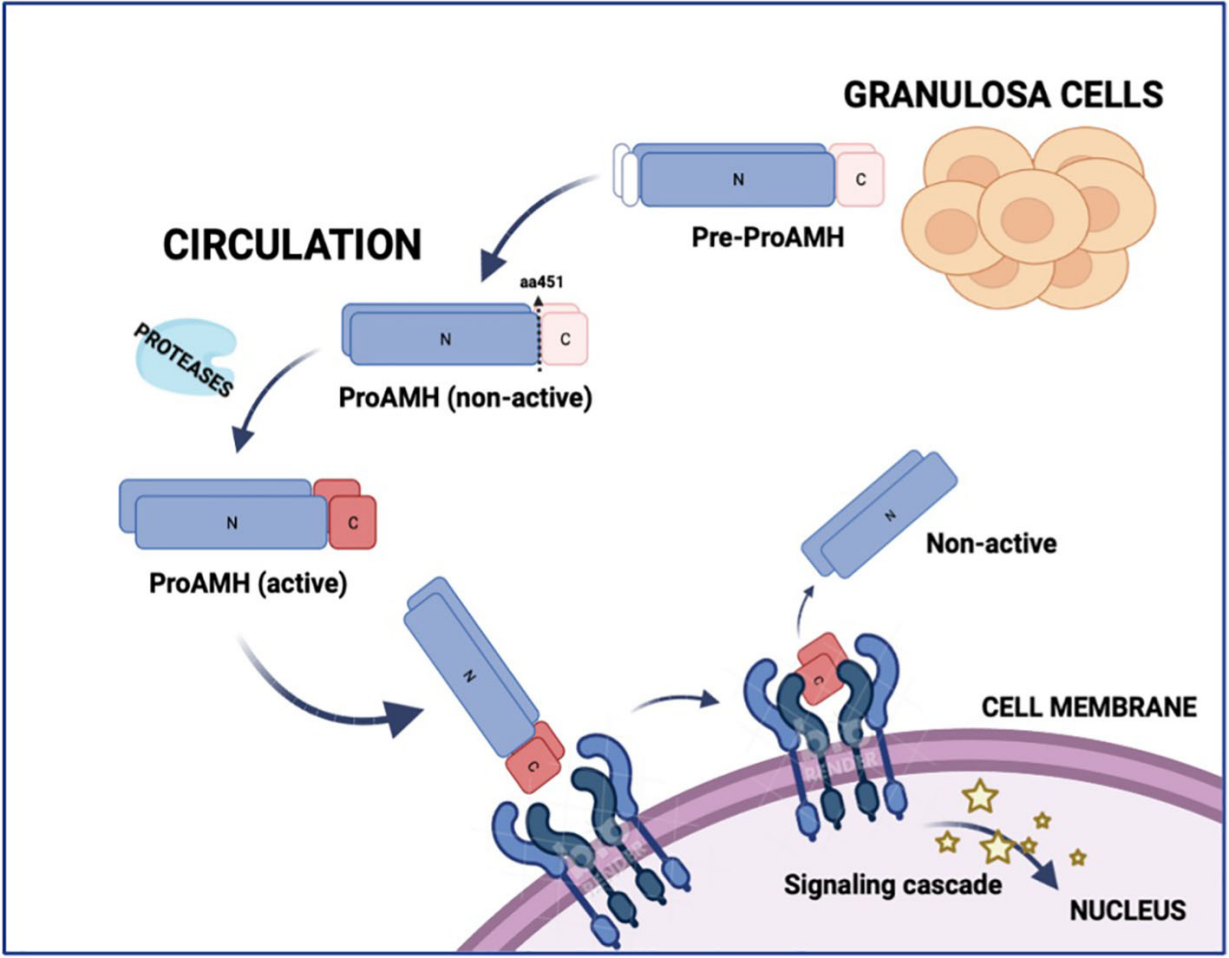
Schematic representation of AMH synthesis, circulation, activation, and receptor binding. Anti-Müllerian hormone (AMH) is synthesized in granulosa cells as a precursor (Pre-proAMH) and secreted into the circulation as proAMH in a non-active form, consisting of an N-terminal pro-region (N) and a C-terminal region (C). Proteolytic cleavage at amino acid 451 generates the biologically active complex (AMH-N,C), in which the N- and C-terminal fragments remain non-covalently associated. The C-terminal fragment binds to AMH receptor type 2 (AMHR2) at the cell membrane, triggering a signalling cascade that activates AMH-responsive genes in the nucleus. N, N-terminal; C, C-terminal.

**Figure 2:**
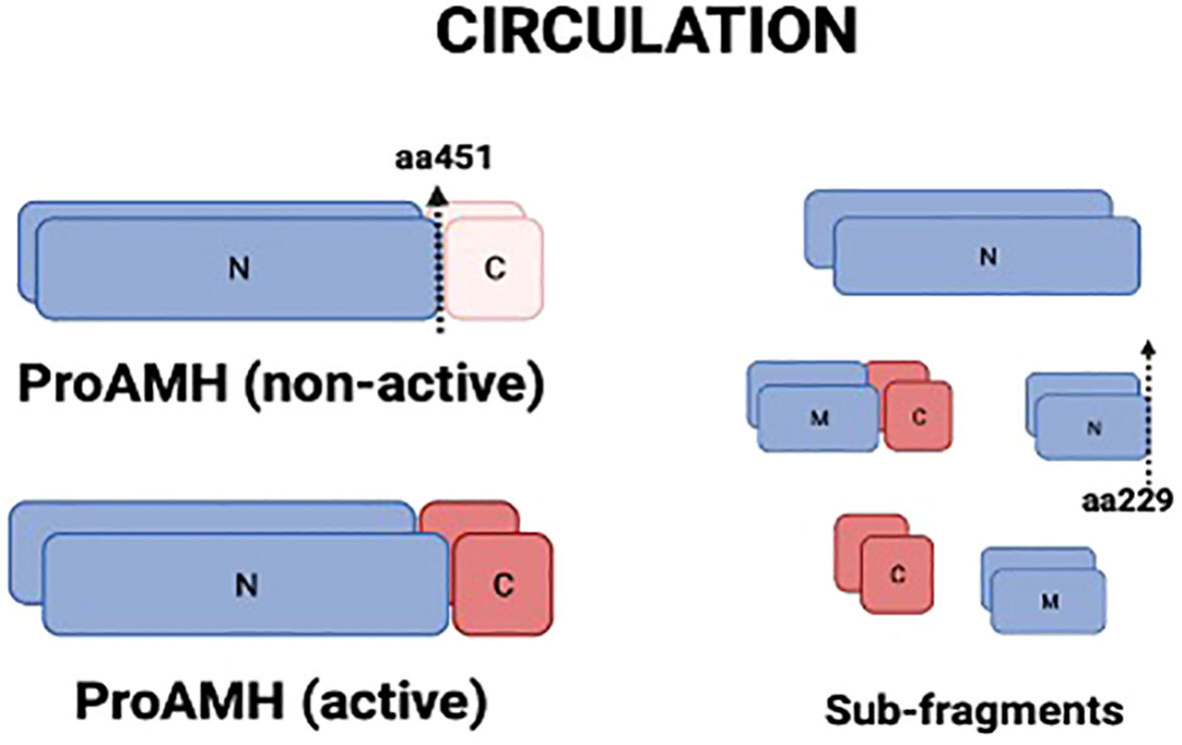
Molecular isoforms of AMH present in circulation. AMH is secreted as proAMH in a non-active form, composed of an N-terminal pro-region (N) and a C-terminal region (C). Proteolytic cleavage at amino acid 451 generates the biologically active complex (proAMH active), in which N- and C-terminal fragments remain associated. Further proteolytic processing at amino acid 229 produces smaller sub-fragments (N, C, and middle fragment, M). Circulating AMH, therefore, consists of a heterogeneous mixture of proAMH, AMH-N,C, and sub-fragments, which may differentially influence assay detection. N, N-terminal; C, C-terminal; M, middle fragment.

Anti-Mullerian hormone (AMH) is a glycoprotein produced by the granulosa cells (GCs) of follicles from the secondary stage onward until the small antral follicle stage, up to 8 mm (4, 5). Functionally, AMH is an essential gatekeeper for the primordial follicle recruitment to the FSH-stimulated antral follicle development, limiting follicle growth initiation (6). The GCs synthesize AMH as an non-active dimeric precursor (pre-proAMH), and it is secreted to the circulation as a non-active protein (proAMH), formed as a N-terminal pro-region (AMH_N_) and a C-terminal pro-region (AMH_C_). This non-active AMH needs proteolytic cleavage at amino acid 451 to become biologically active (AMH_N,C_) and to undertake receptor binding with AMH-R_2_, which starts the cytoplasmic signaling cascade, enters the nucleus and turns on AMH-responsive genes (**Figure 1**). Additional proteolytic processing may take place at amino acid 229 (**Figure 2**).

Circulating AMH is a mixture of different isoforms (proAMH, AMH_N,C_ and other sub-fragments after proteolytic processing) after proteolysis (**Figure 2**), targeted by the monoclonal antibodies in the AMH assays commonly used in clinical practice. Hence, current assays provide an aggregate measure of the two AMH species (proAMH, AMH_N,C_) and miss out on the measurement of AMH_N_ molecules. Those antibodies cannot discriminate between the different circulating isoforms, which might affect the hormone’s quantification (7). The relative levels of proAMH and AMH_N,C_ vary between individuals (Pankhurst et al., 2016a,b), creating platform-specific variation whenever the two species are not equivalently detected. This appears to be a minor issue when estimating ovarian reserve. However, knowledge of the relative levels of proAMH, AMH_N_, AMH_C,_ and AMH_N,C_ is essential for understanding how AMH influences biological processes such as the responsiveness of follicles to FSH.

The recent development of novel high-affinity enzyme-linked immunoabsorbent assays (ELISAs) for AMH, including antibodies directed towards specific epitopes on AMH_C_ or AMH_N_ regions, has improved the accuracy of the assays, providing more reliable results (8, 9). Patients with low ovarian reserve and expected low response to OS may benefit the most from these high-specific tests, as the response to OS may vary significantly from one patient to another. Nevertheless, no previous studies have compared the accuracy of those assays in this sub-group of patients.

The present study aimed to determine whether using different AMH assays, including antibodies to various regions of the AMH molecule, would improve the prediction of the number of oocytes retrieved after OS in patients with poor ovarian reserve.

MATERIALS AND METHODS

## 2 Study design

This prospective observational study was designed to measure AMH using five different AMH assays in patients with low ovarian reserve. A total of 72 women with primary or secondary infertility were included before starting OS for IVF/ICSI at a tertiary referral center for reproductive medicine from February 2019 to December 2021. Low ovarian reserve was defined as AMH serum levels <1.1ng/ml using Elecsys^®^ assay (Roche), following Bologna criteria (10). One Elecsys serum AMH test was performed to define eligible participants during the initial clinical assessment, and patients with AMH Elecsys results <1.1ng/mL were offered to participate in the study. The study serum samples for hormonal analysis were collected the same day the participant started ovarian stimulation; hence, all the hormones included in the study were analyzed using the same serum sample. Ethical approval was obtained (Research Ethics Committee - REFA033c), and the study was registered at Clinicaltrials.gov (NCT03826888). All participants signed informed consent.

## 3 Participants/materials, setting, methods

Participants were excluded if they were pregnant, breastfeeding, smoking, body mass index (BMI) <18 or >35 kg/m2, intake of oral contraceptive pills or any other hormonal treatment during the two previous menstrual cycles before study measurements, endometriosis, any previous medical condition or surgical intervention which could have an impact on the ovarian reserve (e.g. ovarian cyst removal, removal of one or both tubes, tubal ligation for sterilization).

On day 2/3 of the menstrual cycle, before initiating the OS, antral follicle count (AFC) and blood specimens were obtained for same-day results for FSH, LH, estradiol, progesterone and AMH with Elecsys assay (Cobas^®^, Roche). Extra serum samples collected at the same time were frozen at -20C for subsequent analysis, using four different high-affinity enzyme-linked immunoabsorbent assays (ELISAs) for AMH: AL-196 (PCOCheck ELISA), AL-124 (picoAMH ELISA), AL-105 (Ultra SensitiveAMH ELISA) and AL-133 (Total Mature-AMH ELISA) (AnshLab). Frozen samples were batched and shipped together to Ansh Labs (445 Medical Center Blvd, Webster, Texas, 77598, United States), maintaining the frozen storage conditions, and were thawed shortly before measurement. To assess AFC, participants underwent transvaginal 2D-sonography (Voluson E8, GE Healthcare, United States) on day 2/3 of the menstrual cycle. Reproductive medicine specialists performed ultrasound scans, and a systematic ultrasound technique for AFC measurement was used to avoid bias through different strategies to minimize inter-observer variation (11). The number of follicles in each ovary was combined to obtain the AFC. The number of 2 to 10mm in diameter antral follicles were counted (11).

Ovarian stimulation was performed using fixed GnRH-antagonist protocols, recFSH (recombinant follicle-stimulating hormone) or HMG (Human Menopausal Gonadotropin) as stimulation medication. A high dosage of the stimulation medication (300–450 IU/day) was chosen according to the low ovarian reserve parameters considered for inclusion (12). From day 5 onwards, the gonadotrophin dose was adjusted according to oestradiol, FSH and progesterone serum levels (13) and follicular development was assessed by transvaginal ultrasound scan. Final oocyte maturation was achieved by administration of 5.000-10.000 IU of hCG for long protocols and, in case of antagonist protocols, either 5.000-10.000 IU of hCG or dual trigger [hCG and 0.3 mg of GnRH agonist (Triptorelin)], as per physician’s criteria. Oocyte retrieval was carried out 34 or 36 hours later.

### 3.1 AMH assays

All serum samples for AMH were obtained on day 2/3 of the cycle before starting ovarian stimulation. One fresh serum sample was analyzed with Elecsys^®^ AMH automated assay (for Cobas 601 platform, Roche^®^) on the same day the blood was drawn. The assay uses conformational epitope antibodies. Imprecision expected from the assay was <5%, as described by the manufacturer; intra-assay and inter-assay coefficient of variation for Elecsys^®^ AMH automated assay has been reported as 0.5 – 1.4% and 0.7 – 1.9%, respectively (14).

**Figure 3:**
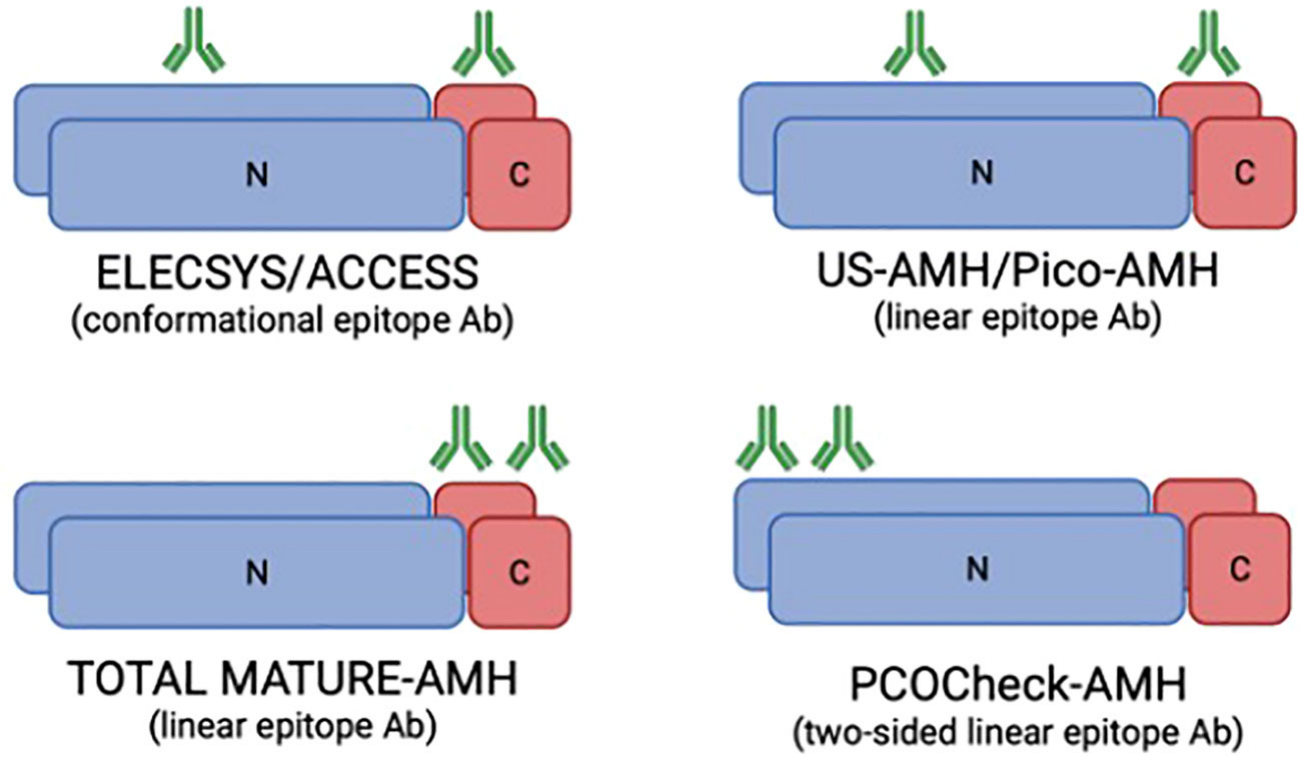
Representation of the monoclonal antibodies binding regions in the AMH molecule for the different AMH ELISA assays used in the study (based on McLennan and Pankhurst, 2015). The Elecsys/Access assays use antibodies directed against conformational epitopes, whereas the US-AMH and Pico-AMH assays recognise linear epitopes. The Total Mature-AMH assay employs linear epitope antibodies detecting multiple isoforms, while the PCOCheck-AMH assay uses a two-sided linear epitope antibody designed to avoid known mutation sites and interferences. Green symbols represent antibody binding sites. Differences in antibody design underlie the variability in assay sensitivity and specificity. N, N-terminal; C, C-terminal; Ab, antibody.

Frozen/thawed serum samples were analyzed at AnshLabs LLC (Webster, TX, USA) using four AMH ELISA assays, using Ansh Labs monoclonal antibody assays against linear epitopes located on the proAMH, AMH_N,C_, AMH_N_, and AMH_C_ regions of AMH (**Figure 3**). AMH assays AL-124 (picoAMH ELISA) and AL-105 (US-AMH ELISA) detect proAMH and AMH_N,C_ (**Table 1**). Assay AL-133 (Total Mature-AMH ELISA) detected proAMH, AMH_N,C_, and AMH_C_ (**Table 1**). AL-196 (PCOCheck AMH ELISA) detected proAMH, AMH_N,C_ and AMH_N_. It uses a two-sided linear epitope antibody with a binding epitope away from the glycosylation sites and AMH mutation sites, with no interference to biotin or follistatin in the sample and high sample stability. The AMH ELISAs used the same standardized recombinant human AMH (cat.: BA047, Ansh Labs, LLC, Webster, TX, USA) calibrators to ensure consistency between assays. Assays have been previously described and validated, and the interassay variations on two serum pools at 70 and 221 pg/mL run over 15 runs were 6.4% and 4.1%, respectively (8, 15, 16).

**Table 1:** AMH isoforms detected by the different ELISA assays.

AMH fragments	PCOCheck ELISA (AL-196)	PicoAMH ELISA (AL-124)	US-AMH ELISA (AL-105)	Total Mature AMH ELISA (AL-133)
ProAMH	√	√	√	√
AMH_N,C_	√	√	√	√
AMH_N_	√	⊗	⊗	⊗
AMH_C_	⊗	⊗	⊗	√

The table shows which molecular isoforms of anti-Müllerian hormone (AMH) are recognized by each assay. ProAMH, pro-hormone form; AMH_N,C_, active complex of N- and C-terminal fragments; AMH_N_, N-terminal fragment; AMH_C_, C-terminal fragment. ✓, detected; ✗, not detected.

### 3.2 Data analysis

Continuous variables are presented as mean (SD) and median with interquartile ranges, while count variables are presented as percentages of the total. The correlation of various AMH kits among themselves and COC metrics was assessed with Spearman’s correlation coefficient. The association of AMH kits and AFC with oocyte yield was modelled with negative binomial regression models. Model performance was evaluated with root mean squared error (RMSE), Nagelkerke R^2^, weighted Akaike Information Criterion, and sigma values. A composite Performance Score metric based on the mentioned performance metrics was used to rank models from best to worst. The performance score was scaled from 0 to 1 so that the worst model always has a score of 0 and the best model always has a score of 1. All analyses were conducted in R using “MASS” and “performance” packages. P values below 0.05 were considered statistically significant.

**Results**

#### 3.2.1 Patient characteristics and stimulation outcomes

The median age of the patients included was 39 years (IQR: 36-42), and their BMI was 28.51Kg/m^2^ (IQR: 26.1-30.1 Kg/m^2^). Following the inclusion criteria, the serum AMH median measured with Elecsys was 0.64 ng/mL (IQR: 0.29-0.81ng/mL), a median AFC of 5.5 (IQR: 4-7) and a basal FSH of 8.23 mIU/mL (IQR: 6.4-12.5 mIU/mL). Patients’ characteristics, the hormonal assessment, and the outcome of the ovarian stimulation cycle are presented in **Table 2**.

**Table 2:** Patient characteristics, AMH serum levels, and stimulation outcomes (n = 72).

n=72	Mean ± Std. Dev.	Min - Max	Median	IQR
Age (years)	38.58±4.93	26.00 – 46.00	39.00	36 - 42
BMI (kg/m^2^)	28.29±3.10	22.36 – 35.01	28.51	26.07 - 30.09
E2 basal (pg/mL)	45.89±28.69	5.00 – 226.00	41.18	28.47 - 56.44
FSH basal (mIU/mL)	10.03±4.91	4.33 – 25.03	8.23	6.4 - 12.5
LH basal (mIU/mL)	6.95±3.24	1.34 – 18.11	6.54	4.51 - 8.91
AFC (n)	5.74±3.31	1.00 – 17.00	5.50	4.0 - 7.0
AMH Elecsys (ng/mL)	0.58±0.30	0.02 -1.05	0.64	0.29 - 0.81
AL-105 (ng/mL)	1.03±0.58	0.05 -2.26	0.89	0.57 - 1.39
AL-196 (ng/mL)	0.72±0.42	0.03 – 1.59	0.64	0.39 - 0.99
AL-124 (ng/mL)	0.91±0.54	0.00 – 2.17	0.79	0.49 - 1.24
AL-133 (ng/mL)	1.13±0.65	0.00 – 2.95	1.06	0.63 - 1.59
Follicles DoT (n)	5.42±3.44	1.00 – 20.00	5.00	3.0 - 7.0
COC (n)	3.74±2.56	1.00 – 14.00	3.00	2 - 4.5
MII (n)	2.94±2.26	0.00 – 13.00	3.00	1.0 - 4.0

Data are presented as mean ± standard deviation (SD), median with interquartile range (IQR), and minimum–maximum values. Variables include basal hormone levels, antral follicle count (AFC), AMH levels measured with different assays (Elecsys, AL-105, AL-196, AL-124, AL-133), and ovarian stimulation outcomes: number of follicles on the day of trigger (DoT), cumulus–oocyte complexes (COCs), and metaphase II oocytes (MII).

Regarding the stimulation outcomes, the median number of follicles on the day of trigger (Fdot) was 5 (IQR: 3-7), the number of retrieved cumulus-oocyte-complexes (COC’s) was 3 (IQR: 2-4.5), and the metaphase II oocytes (MII) were 3 (IQR: 1-4) (**Table 2**).

#### 3.2.2 Correlation between AMH assays, AFC and ovarian stimulation outcomes

**Figure 4:**
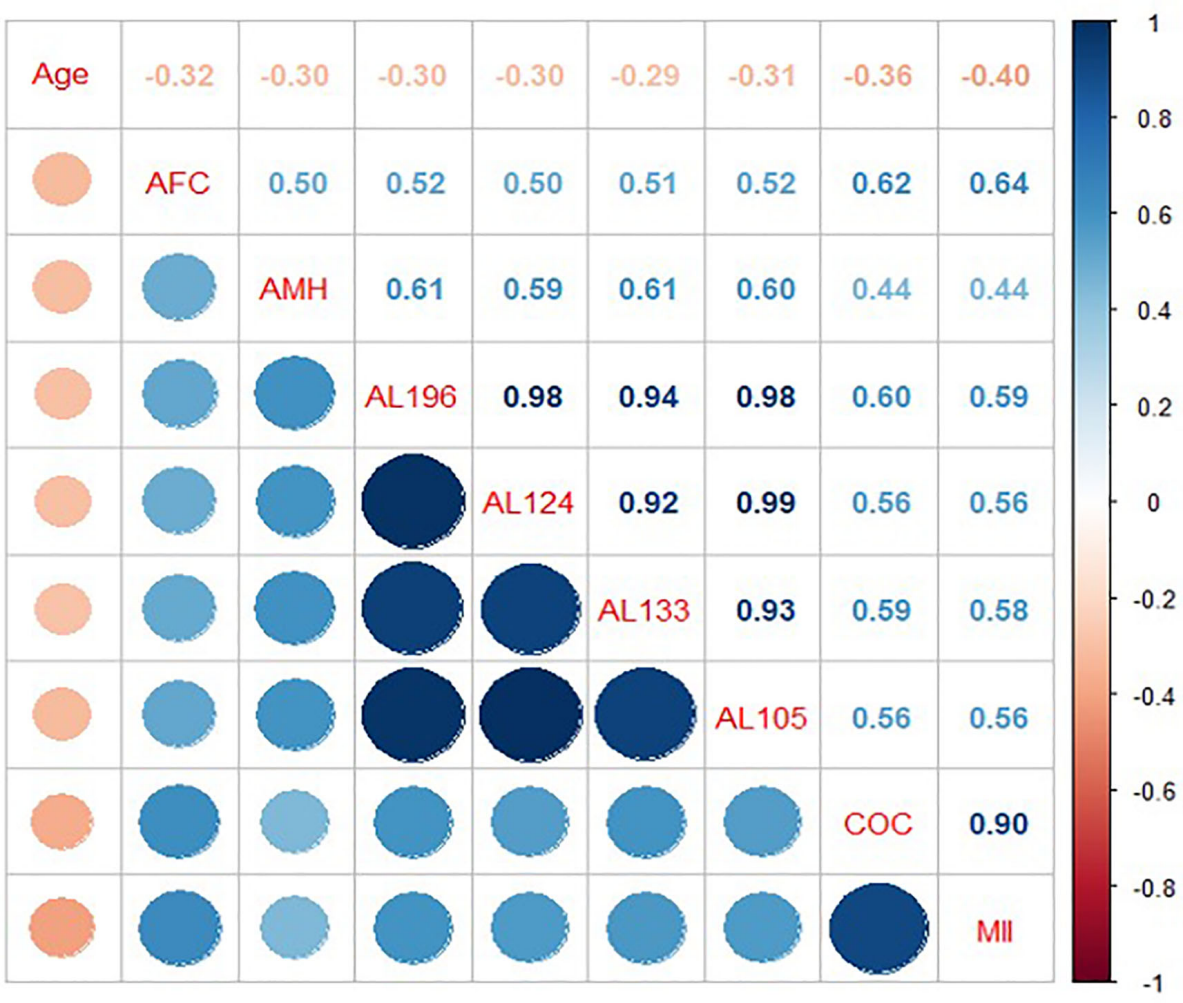
Correlation matrix of age, antral follicle count (AFC), AMH assays, and ovarian stimulation outcomes. The matrix is based on Spearman correlation coefficients (values shown within each cell; coefficients range from –1 to +1). Positive correlations are indicated by blue shades, negative correlations by red/orange shades, with the intensity of the colour and circle size proportional to correlation strength (scale shown on the right). AMH refers to Elecsys AMH ELISA assay performed on fresh serum; AL-196 (PCOCheck ELISA), AL-124 (picoAMH ELISA), AL-133 (Total Mature-AMH ELISA), and AL-105 (US-AMH ELISA) were performed on frozen serum. Variables include age, AFC, cumulus–oocyte complexes retrieved (COCs), and metaphase II oocytes (MII). All correlations were statistically significant (p < 0.05).

AMH serum levels measured with Elecsys assay is the standard routine test used in our clinical practice, and it revealed a good correlation with AFC (rs=0.50, *p*<0.043) (**Figure 4**; **Supplementary Table 1**). When considering the AMH assays performed by AnshLabs (AL-133, AL-124, AL-196 and AL 105), their results presented a high consistency between them (*p*<0.001) (**Figure 4**). All AMH assays significantly correlated with COCs and MII (**Supplementary Table 2**). However, AL-196 showed the highest correlation (rs=0.60, rs=0.59, respectively; *p*<0.001) (**Figure 4**).

#### 3.2.3 Comparative performance for total COCs collected with the different AMH assays and AFC

**Figure 5:**
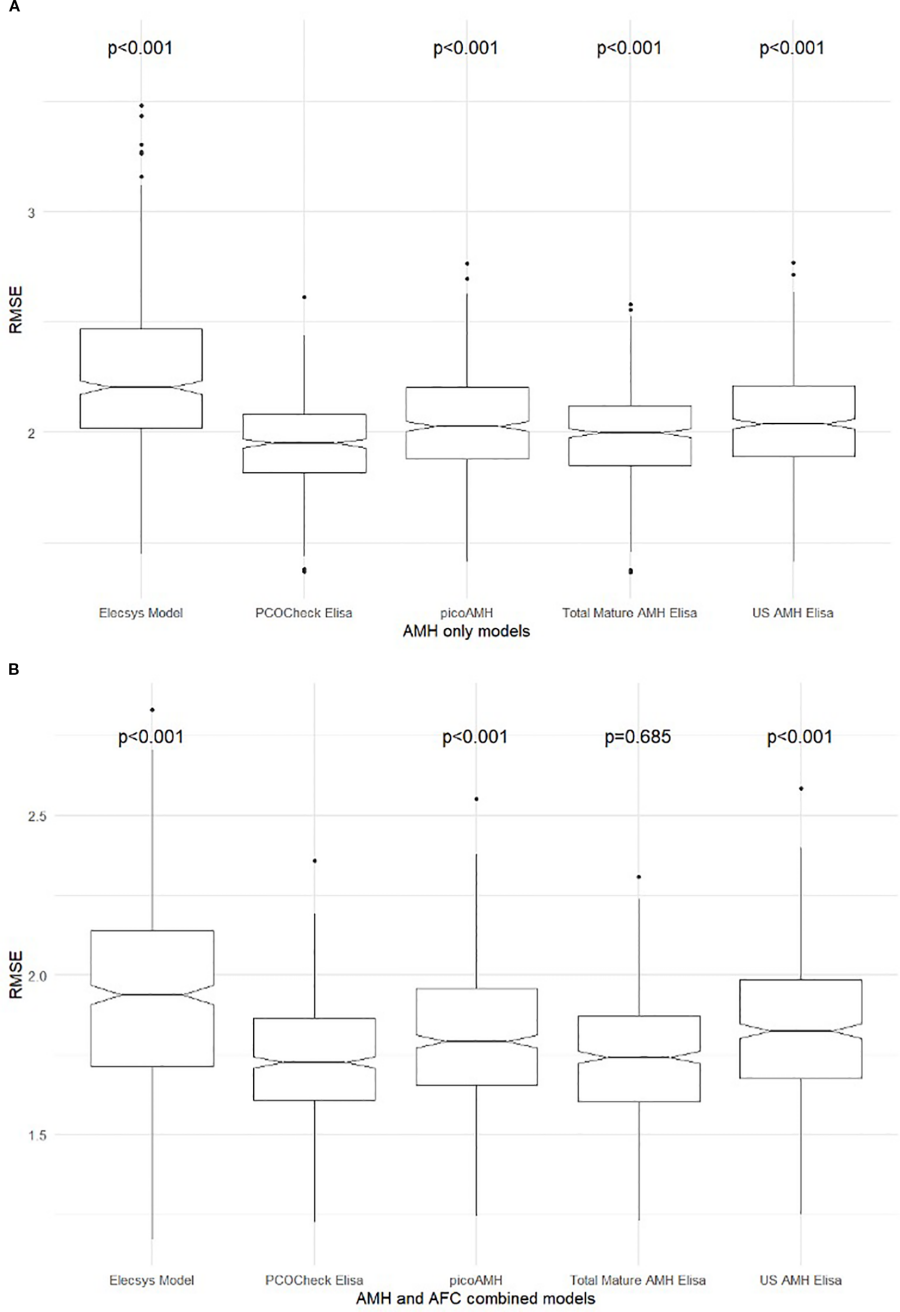
Root mean square error (RMSE) results comparing the predictive performance of different AMH assays. **(A)** RMSE distribution for AMH-only models. **(B)** RMSE distribution for combined models including both AMH assay results and antral follicle count (AFC). Each boxplot represents the model’s predictive accuracy for cumulus–oocyte complexes (COCs) retrieved after ovarian stimulation. Lower RMSE values indicate better predictive performance. Statistical comparisons were performed against AL-196 (PCOCheck ELISA). AMH = Elecsys AMH ELISA (Roche, fresh serum); AL-196 = PCOCheck AMH ELISA; AL-124 = picoAMH ELISA; AL-133 = Total Mature AMH ELISA; AL-105 = US-AMH ELISA (all performed on frozen serum). All p-values are shown above each boxplot.

After ovarian stimulation, the range of COCs collected was 1 to 14 (**Table 2**). To evaluate the accuracy of the different tests for predicting the number of COCs collected, Root Mean Square Error (RMSE) was used, considering different models: (i) including only AMH assays (**Figure 5A**), and (ii) combining AMH performed with different assays plus AFC (**Figure 5B**) (**Tables 3A**, **B**; **Supplementary Figures 1**, **2**). The lower RMSE results were observed when using AMH and AFC combined models, indicating a better fit of the model to the actual number of COCs retrieved (**Tables 3A**, **3B**). AL-196 (PCOCheck Elisa) showed the best accuracy among the models tested (*p*<0.001). Yet, no statistical differences were found when AL-196 was compared to AL-133 (Total Mature AMH Elisa) in the AMH+AFC combined model (*p* = 0.685) (**Figure 5B**). Although no statistical difference was observed between AL-196 and AL-133 in the AMH+AFC combined model, AL-196 consistently showed superior performance across multiple metrics (RMSE, Performance Score, AIC weight).

**Table 3A:** Root mean square error (RMSE) of AMH-only models.

RMSE ANALYSIS	Elecsys	PCOCheck ELISA (AL-196)	Total Mature AMH ELISA (AL-133)	US-AMH ELISA (AL-105)	Pico AMH ELISA (AL-124)
RMSE using AMH-only models [mean value (range)]	2.20 (2.02 to 2.47)	1.95 (1.82 to 2.08)	2.00 (1.85 to 2.12)	2.04 (1.89 to 2.21)	2.03 (1.88 to 2.20)

Each model included one AMH assay (Elecsys, PCOCheck AMH [AL-196], Total Mature AMH [AL-133], US-AMH [AL-105], or picoAMH [AL-124]) to predict cumulus–oocyte complex (COC) yield. Lower RMSE values indicate better predictive accuracy. Results are shown as mean values with ranges.

**Table 3B:** Root mean square error (RMSE) of models combining AMH assays with antral follicle count (AFC).

RMSE ANALYSIS	Elecsys	PCOCheck ELISA (AL-196)	Total Mature AMH ELISA (AL-133)	US-AMH ELISA (AL-105)	Pico AMH ELISA (AL-124)
RMSE using AMH+AFC models [mean value (range)]	1.94 (1.71 to 2.14)	1.73 (1.61 to 1.87)	1.74 (1.60 to 1.87)	1.82 (1.68 to 1.99)	1.79 (1.66 to 1.96)

Each model included one AMH assay plus AFC as predictors of cumulus–oocyte complex (COC) yield. Lower RMSE values indicate better predictive accuracy. Results are shown as mean values with ranges.

#### 3.2.4 Performance score of the different models

Additionally to RMSE, further performance metrics described in **Table 4** were used to evaluate both models. AL-196 (PCOCheck Elisa) consistently presented the best Performance Score for AMH-only models and for AMH plus AFC models (**Table 4**).

**Table 4:** Model performance metrics for AMH-only and AMH+AFC combined models.

Variables	Nagelkerke R^2^	RMSE	Sigma	AIC wt	Performance Score
Models including AMH assays only					
• AL-196 (PCOCheck)	0.57	2.00	0.98	0.58	1.00
• AL-133 (Total Mature)	0.56	2.05	0.99	0.34	0.82
• AL-124 (Pico AMH)	0.51	2.10	1.02	0.04	0.53
• AL-105 (US-AMH)	0.51	2.11	1.02	0.04	0.52
• Elecsys	0.38	2.29	1.10	0.00	0.00
Models including AMH assays and AFC					
• AFC + AL-196 (PCOCheck)	0.68	1.80	0.89	0.43	0.99
• AFC + AL-133 (Total Mature)	0.68	1.80	0.89	0.39	0.97
• AFC + AL-124 (Pico AMH)	0.66	1.89	0.92	0.11	0.56
• AFC + AL-105 (US-AMH)	0.65	1.91	0.92	0.07	0.46
• AFC + Elecsys	0.60	2.01	0.97	0.00	0.00

Performance was evaluated using Nagelkerke R², RMSE (root mean square error), sigma, Akaike Information Criterion weight (AIC wt), and a composite Performance Score (scaled from 0 to 1, with the best model set to 1.00). Models included AMH-only assays (Elecsys, AL-196 [PCOCheck], AL-133 [Total Mature], AL-124 [picoAMH], AL-105 [US-AMH]) and AMH+AFC combined models.

## 4 Discussion

Predicting the number of oocytes which might be retrieved after OS is particularly challenging in patients with low ovarian reserve. The expected number of oocytes might directly impact the decision-making process for undergoing an IVF/ICSI treatment. Besides, after OS, a wide range of oocytes might be expected for patients with serum AMH levels below 1.1ng/mL (COCs min-max 1-14, MIIs 0-13; **Table 2**), and a reliable way to improve the prediction of the retrieved oocytes would be very useful for daily clinical practice. This study demonstrates that high-affinity assays for AMH, including antibodies directed towards specific epitopes on AMH_C_ and AMH_N_ regions, improve the prediction of oocytes collected after OS for patients with low ovarian reserve. While it is true that the differences in correlation coefficients between the assays appear modest, their clinical significance lies in the context of patients with low ovarian reserve. For women with diminished reserve, where every retrieved oocyte is critical, even small improvements in predictive models can influence clinical decisions. While the Elecsys assay remains a robust tool, our findings suggest that isoform-targeting assays, particularly AL-196, offer an incremental benefit, which can translate into meaningful improvements in outcomes and patient care.

AMH’s clinical utility ranges from a marker of testicular function to the assessment of ovarian reserve, a variety of ovarian diseases, oncofertility and gonadotoxicity, which has increased the need for highly sensitive and specific tests (17). However, the presence of different AMH isoforms complicates the accuracy of the measurement, and the re-design of capture and detection antibodies for AMH immunoassays has arisen (9). The proteolysis of the pro-AMH (the precursor hormone) generates a 58 kDa N-terminal domain (AMH_N_) and a biologically active 12.5 kDa C-terminal domain (AMH_C_) (18). Commercial AMH assays target various parts of the AMH hormone with assay-specific antibodies and target the mature region, the pro-region or both (**Figure 3**). In a cross-sectional study comparing AMH levels among three commercially available AMH immunoassays (AMH Gen II, Beckman Coulter; US AMH (AL-105), AnshLab; and picoAMH (AL-124), AnshLab), significantly higher proportions of detectable AMH levels were observed with the picoAMH assay (97%) and US-AMH assay (92%) (AnshLab) compared to Gen II assay (84%) (19). The different antibody selection used for the AnshLab ELISA tests (pico-AMH and US-AMH) compared to Gen II assay may contribute to the observed differences. Moreover, AMH epitopes might be masked by protein interaction in the circulation. Hence, the continued development of antibody design for ELISAs for glycoprotein hormones must consider variations in specificity, cross-reactivities, epitope locations (20) and clinical application.

Recently, a novel ELISA assay, PCOCheck (AL-196, AnshLabs), has been developed, which uses a linear epitope two-sided antibody specifically designed to avoid antibody binding to known AMH mutation sites. Additionally, based on its ability to bind the epitope in its linear configuration, the results are not impacted by conformational changes due to thermal instability nor interferences to biotin or follistatin in the sample (21). In the present study, the PCOCheck assay showed the highest Performance Score when evaluating models including only AMH and AMH plus AFC models (**Table 4**). The other assessed assays in the herein study, including linear epitope antibodies [Total Mature AMH ELISA (AL-133), US AMH ELISA (AL-105) and Pico AMH ELISA (AL-133)], presented, as well, higher Performance Scores compared to Elecssys, which includes conformational epitope antibodies (22).

These considerations regarding the antibodies in the AMH ELISA assays help to underscore their importance for everyday clinical practice when evaluating women’s ovarian reserve. Antral follicle count (AFC) and AMH levels are considered the best markers for functional ovarian reserve assessment, and several publications have demonstrated a strong positive correlation between them (1), with similar fluctuations throughout the menstrual cycle (23, 24). Nevertheless, the frequent lack of alignment between AMH and AFC in forecasting the number of COCs and MII retrieved post-ovarian stimulation is a common concern for clinicians, with variations that can span from minor to clinically significant. Some extreme examples are the cases where severely reduced serum AMH levels are found in patients with high AFC (25–27). In the present study, the prediction of the number of COCs and MII collected for patients previously diagnosed with low ovarian reserve was improved when AMH ELISA assays included high-specific antibodies (higher Performance Score, **Table 4**). It is worth mentioning that the combination of AMH plus AFC improved the prediction compared to using AMH as a single marker, in line with previous publications (28), which will help patients and clinicians for counselling and decision-making before starting ovarian stimulation and to anticipate cases with extremely poor response.

The present study prospectively evaluated five different AMH ELISA assays, four of them incorporating high-specific antibodies, and demonstrated both the high affinity of these antibodies and the improvement in assay performance. While all clinical assessments were performed at the same center following standardized methodology, inter-observer variability in AFC remains a potential limitation. Although no statistical difference was observed between AL-196 (PCOCheck) and AL-133 (Total Mature AMH ELISA) in the AMH+AFC combined model, AL-196 consistently demonstrated superior performance across multiple evaluation metrics, including RMSE, Performance Score, and AIC weight. This trend supports its potential clinical advantage; however, we acknowledge that the limited sample size may have reduced the statistical power to detect subtle differences, and larger cohorts will be required to confirm and strengthen these findings. Another methodological consideration is that although all AMH assays were performed on the same serum sample collected on the same day, fresh serum was used for the Elecsys assay, whereas frozen aliquots were shipped to Ansh Labs (Texas) for batched analysis. While this could be regarded as a limitation, prior studies have demonstrated the high stability of AMH during freeze–thaw processes (29), and results obtained with linear epitope antibody assays should not be affected by freezing or conformational changes in the protein. Importantly, our design—using a single blood sample per patient, split for fresh and frozen analyses—ensures comparability across assays. Thus, the AMH-only models provide not only a comparison between assays but also a direct evaluation of fresh (Elecsys) versus frozen (AnshLabs) samples, further supporting the robustness of our findings. Finally, a limitation of this study is that AMH isoforms and immune biomarkers were not quantified in healthy women for comparison with patients of low ovarian reserve, which represents an important direction for future research.

## 5 Conclusion

In patients with low ovarian reserve, combining AFC and highly specific AMH assays using linear-epitope antibodies, such as PCOCheck (AL-196), enhances the accuracy of predicting the number of oocytes retrieved. Since patients with serum AMH levels below 1.1 ng/mL may exhibit a broad range of oocyte yields following ovarian stimulation, these advanced AMH assays, when combined with AFC, offer improved prediction of clinical outcomes and better anticipation of very poor responses.

## Data Availability

The raw data supporting the conclusions of this article will be made available by the authors, without undue reservation.

## Supplementary material

Supplementary Figure 1 Correlation of predicted and actual cumulus–oocyte complexes (COCs) retrieved using combined models (AFC plus one AMH assay). Each panel shows the performance of a different AMH assay in combination with antral follicle count (AFC): Elecsys (red), PCOCheck ELISA (blue), picoAMH ELISA (green), Total Mature AMH ELISA (purple), and US AMH ELISA (orange). The black line represents the perfect fit (identity line), the dashed line indicates the observed regression fit, and the shaded area represents the 95% confidence interval for the fit.

Supplementary Figure 2 Correlation of predicted and actual cumulus–oocyte complexes (COCs) retrieved using models including only one AMH assay. Each panel shows the performance of a different AMH assay: Elecsys (red), PCOCheck ELISA (blue), picoAMH ELISA (green), Total Mature AMH ELISA (purple), and US AMH ELISA (orange). The black line represents the perfect fit (identity line), the dashed line shows the observed regression fit, and the shaded area indicates the 95% confidence interval for the fit.
