# Supplementary figures and images for: How to enhance prediction of clinical outcomes in poor responders: integrating high-specific assays for anti-mullerian hormone with antral follicle count
Source: Front Endocrinol (Lausanne). 2025 Sep 29;16:1654365. doi: 10.3389/fendo.2025.1654365 (PMC12515637; doi:10.3389/fendo.2025.1654365)

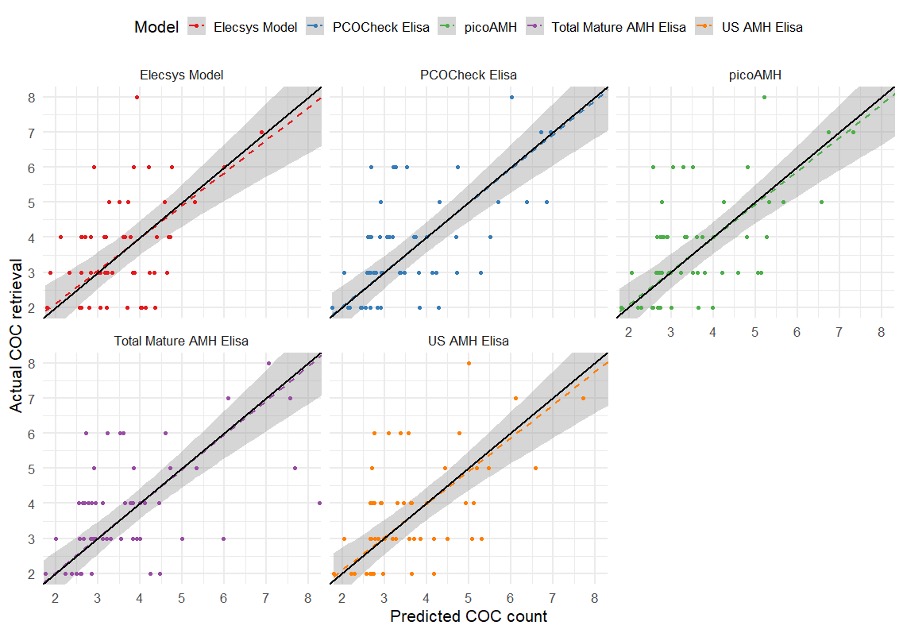

Supplement: Supplementary Figure 1 — Correlation of predicted and actual cumulus–oocyte complexes (COCs) retrieved using combined models (AFC plus one AMH assay). Each panel shows the performance of a different AMH assay in combination with antral follicle count (AFC): Elecsys (red), PCOCheck ELISA (blue), picoAMH ELISA (green), Total Mature AMH ELISA (purple), and US AMH ELISA (orange). The black line represents the perfect fit (identity line), the dashed line indicates the observed regression fit, and the shaded area represents the 95% confidence interval for the fit. [file Image1.jpeg]

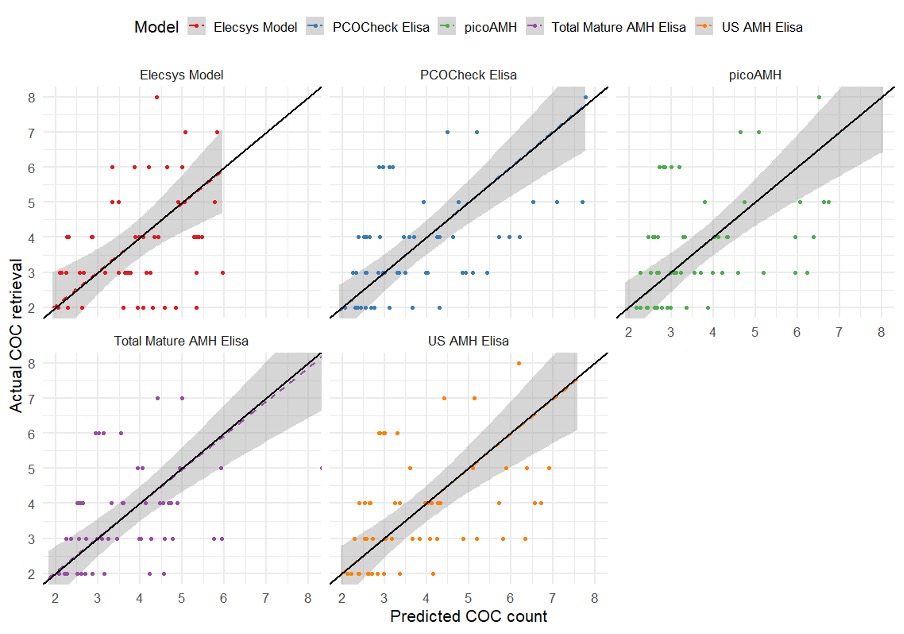

Supplement: Supplementary Figure 2 — Correlation of predicted and actual cumulus–oocyte complexes (COCs) retrieved using models including only one AMH assay. Each panel shows the performance of a different AMH assay: Elecsys (red), PCOCheck ELISA (blue), picoAMH ELISA (green), Total Mature AMH ELISA (purple), and US AMH ELISA (orange). The black line represents the perfect fit (identity line), the dashed line shows the observed regression fit, and the shaded area indicates the 95% confidence interval for the fit. [file Image2.jpeg]
