# Supplementary material for: How to enhance prediction of clinical outcomes in poor responders: integrating high-specific assays for anti-mullerian hormone with antral follicle count
Source: Front Endocrinol (Lausanne). 2025 Sep 29;16:1654365. doi: 10.3389/fendo.2025.1654365 (PMC12515637; doi:10.3389/fendo.2025.1654365)
Supplement: Supplementary file 3 [file Table1.docx]

**Supplementary data**

Supplementary Table 1: Spearman correlation between AMH assays with AFC.

| Variables | *mean* | *Low CI* | *Up CI* | *p* |
| --- | --- | --- | --- | --- |
| Elecsys AMH | 0.499 | 0.032 | 0.926 | 0.043 |
| AL196 | 0.518 | -0.065 | 0.911 | 0.071 |
| AL124 | 0.495 | -0.103 | 0.905 | 0.086 |
| AL133 | 0.510 | -0.058 | 0.912 | 0.069 |
| AL105 | 0.514 | -0.081 | 0.908 | 0.077 |

Supplementary Table 2: Spearman correlation between AMH assays and number of follicles on the day of trigger (Fdot), COCs and MII. *All correlations *p*<0.001.

| Spearman correlation* | Fdot | COCs | MII |
| --- | --- | --- | --- |
| Elecsys AMH | 0.61 | 0.50 | 0.49 |
| AL105 | 0.62 | 0.58 | 0.59 |
| AL196 | 0.62 | 0.59 | 0.60 |
| AL124 | 0.61 | 0.58 | 0.59 |
| AL133 | 0.57 | 0.57 | 0.57 |
